# Embedding electron-deficient nitrogen atoms in polymer backbone towards high performance n-type polymer field-effect transistors†
†Electronic supplementary information (ESI) available. See DOI: 10.1039/c6sc01380e


**DOI:** 10.1039/c6sc01380e

**Published:** 2016-06-13

**Authors:** Ya-Zhong Dai, Na Ai, Yang Lu, Yu-Qing Zheng, Jin-Hu Dou, Ke Shi, Ting Lei, Jie-Yu Wang, Jian Pei

**Affiliations:** a Beijing National Laboratory for Molecular Sciences , Key Laboratory of Bioorganic Chemistry and Molecular Engineering of Ministry of Education , Key Laboratory of Polymer Chemistry and Physics of Ministry of Education , Center for Soft Matter Science and Engineering , College of Chemistry and Molecular Engineering , Peking University , Beijing 100871 , China . Email: jianpei@pku.edu.cn

## Abstract

With sp^2^-nitrogen atoms embedded in an isatin unit, a donor–acceptor (D–A) conjugated polymer **AzaBDOPV-2T** was developed with a low LUMO level down to –4.37 eV. The lowered LUMO level as well as the conformation-locked planar backbone provide **AzaBDOPV-2T** with electron mobilities over 3.22 cm^2^ V^–1^ s^–1^ tested under ambient conditions, which is among the highest in n-type polymer field-effect transistors (FETs). Our results demonstrate that embedding electron-deficient sp^2^-nitrogen in conjugated backbones is an effective approach to develop n-type polymer semiconductors with high performance.

## Abstract 2

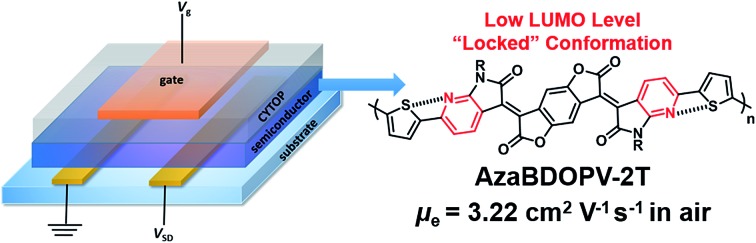
The low LUMO level and the conformation-locked planar backbone provide polymer **AzaBDOPV-2T** with electron mobilities over 3.22 cm^2^ V^–1^ s^–1^ tested in air.

## 

Conjugated polymers have attracted great interests in low-cost, flexible, and large-area electronic applications due to their solution-processability, good mechanical properties, and tunable electronic properties.^1^ In the past few years, the development of novel building blocks for conjugated polymers, such as benzothiadiazole (BT),^2^ diketopyrrolopyrrole (DPP),^3^ isoindigo (II),^4^ and naphthalene diimide (NDI),^5^ has led to significant progress in the carrier mobilities of polymer semiconductors. Nevertheless, only a few of these polymers can exhibit high electron mobilities when operated under ambient conditions,5*c* thus limiting their application. It has been an intriguing research topic to develop high-performance air-stable n-type polymer semiconductors in organic electronics.

Low LUMO levels are indeed desirable to facilitate electron injection and enhance the electrochemical stability of n-type polymer semiconductors.^6^ The introduction of electron-withdrawing groups (such as F or Cl) onto polymer conjugated backbones has been shown to be an effective way to lower their LUMO energy levels, thus leading to enhanced n-type transport.6c,d However, compared to the extensive efforts spent on halogen substitution, embedding electron-deficient sp^2^-nitrogen atoms in polymer backbones has been somewhat overlooked.^7^ Incorporation of electron-deficient pyridine units into polymer backbones could also lower the energy levels of the resultant conjugated polymers. In addition, the replacement of benzene rings with pyridine units is also beneficial to improving the planarity of polymer backbones because sp^2^-nitrogen atoms are less sterically demanding than CH units.^8^ Therefore, such a sp^2^-nitrogen embedding strategy may provide new opportunities to develop decent n-type semiconductors.

Recently, benzodifurandione-based oligo(*p*-phenylene vinylene) (**BDOPV**), which has four electron-withdrawing carbonyl groups on the backbone and a low LUMO level of –4.24 eV, was developed as an excellent electron-accepting moiety for a large number of semiconductors in field-effect transistors (FETs).^9^ Specially, the D–A conjugated polymer **BDOPV-2T** showed a high electron mobility up to 1.74 cm^2^ V^–1^ s^–1^.9*c* To further improve the electron transport property of **BDOPV**-based polymers, herein, we embed sp^2^-nitrogen atoms in **BDOPV**, resulting in a stronger electron-deficient building block diaza-**BDOPV** (**AzaBDOPV**) (Fig. 1). The D–A conjugated polymer **AzaBDOPV-2T** shows a more planar backbone and a lower LUMO level (down to –4.37 eV) compared with **BDOPV-2T**. As a consequence, **AzaBDOPV-2T** exhibits higher electron mobilities of over 3.22 cm^2^ V^–1^ s^–1^ for devices tested under ambient conditions, which is among the highest in n-type polymer FETs.5c,^10^

**Fig. 1:**
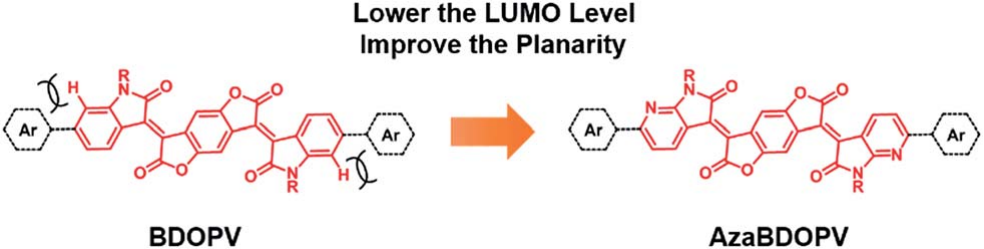
Molecular structures of **BDOPV** and **AzaBDOPV**.

To achieve the monomer **AzaBDOPV**, 6-bromo-7-azaisatin was synthesized from a commercial available precursor, 7-azaindole.^11^ As illustrated in Scheme 1, 7-azaindole (**1**) was converted into *N*-oxide-7-azaindole (**2**) by *m*-CPBA in high yield. Benzoyl bromide was used as the brominating reagent to afford a Reissert–Henze salt.^12^ Hence, the bromination reaction only occurred at the *ortho*-position relative to the nitrogen atom on pyridine. The benzoyl group on the nitrogen atom of the pyrrole was subsequently removed by the base treatment, giving 6-bromo-7-azaindole (**3**) in 56% yield for the two steps. Then, the alkylation of **3** gave the corresponding alkylated azaindole (**4**) in 82% yield, which was further oxidated through pyridinium chlorochromate (PCC) to afford 6-bromo-7-azaisatin in 68% yield.^13^ Interestingly, 6,6′-dibromo-7,7′-azaisoindigo was also observed in 10% yield in this oxidation reaction.13*a* Further investigation revealed that 6,6′-dibromo-7,7′-azaisoindigo was afforded exclusively in 36% yield when using dry PCC and excess AlCl_3_. The generation of azaisoindigo was expected as a result of the Lewis-acid-catalyzed aldol condensation between the azaisatin and azaindole, with subsequent dehydration and oxidation as shown in Fig. S1.† An excess amount of AlCl_3_ and a non-water environment may promote this process, resulting in selectively affording compound **6**.13*b* Considering the potential optoelectronic applications of azaisoindigo derivatives, our method provides a shorter and simpler strategy to obtain azaisoindigo compared with the more common method that requires the coupling of oxindole with isatin.

**Scheme 1:**
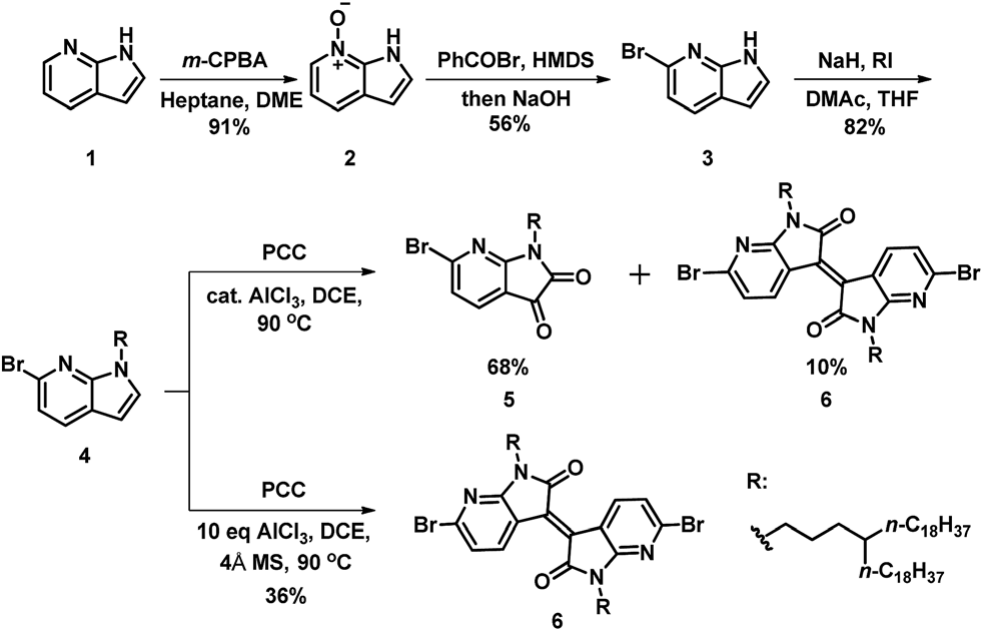
Synthetic route to 6-bromo-7-azaisatin and its derivatives.

As shown in Scheme 2, **AzaBDOPV** was synthesized in 40% yield by a condensation reaction between **5** and benzo[1,2-*b*:4,5-*b*′]difuran-2,6(3*H*,7*H*)-dione in the presence of 4-methylbenzenesulfonic acid. Finally, a Stille coupling polymerization reaction between **AzaBDOPV** and 5,5′-bis(trimethylstannyl)-2,2′-bithiophene gave the desired polymer **AzaBDOPV-2T** in 90% yield after purification (see the ESI†). **AzaBDOPV-2T** displayed a high molecular weight with an *M*_n_ of 51.6 kDa and a polydispersity index (PDI) of 2.62, which was evaluated by high temperature gel permeation chromatography at 150 °C using 1,2,4-trichlorobenzene as the eluent (Fig. S2†). **AzaBDOPV-2T** also showed excellent thermal stability with a decomposition temperature of over 390 °C (Fig. S3†).

**Scheme 2:**
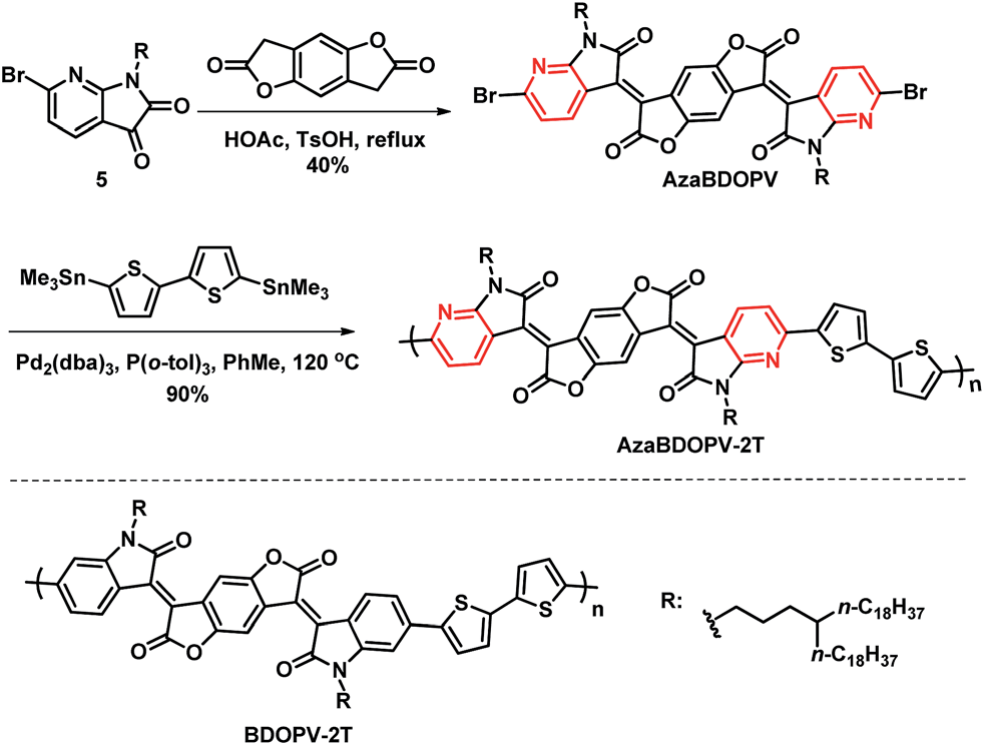
Synthesis of polymer **AzaBDOPV-2T** and structure of the reference polymer **BDOPV-2T**.

The absorption spectra of the polymer **BDOPV-2T** in dilute solution, and the polymer **AzaBDOPV-2T** both in dilute solution and in a thin film, are shown in Fig. 2. The bandgap of **AzaBDOPV-2T** calculated from the onset of film absorption is 1.32 eV. **AzaBDOPV-2T** displayed a dual-band absorption both in solution and in film with obvious 0–0 and 0–1 vibrational peaks. Compared with the absorption of **BDOPV-2T** in dilute solution, the 0–0 vibrational peak of **AzaBDOPV-2T** was clearly observed in solution, indicating that **AzaBDOPV-2T** has a more planar backbone structure relative to **BDOPV-2T**. No significant redshift of the absorption was observed for **AzaBDOPV-2T** from the solution to the film, which indicates that the polymer probably forms some preaggregates in solution due to strong intermolecular interactions. Interestingly, computational analysis of the **AzaBDOPV-2T** fragment reveals that the sulfur and pyridinic nitrogen atoms were favorable to be on the same side. The calculated S–N distance (2.27 Å) is significantly shorter than the sum of the S–N van der Waals radii (3.35 Å), indicating a typical S–N interaction,^14^ which may “lock” the polymer conformation. As a result, the calculated pyridyl–thienyl rotational angle in **AzaBDOPV-2T** is decreased from 21.5° to 0.5°, revealing that the introduction of nitrogen atoms provides the polymer with a more planar backbone and better π-conjugation, which is consistent with the absorption features.

**Fig. 2:**
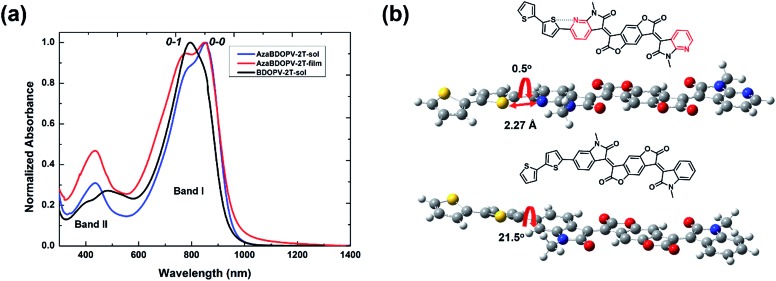
(a) The absorption spectra of the polymer **BDOPV-2T** in dilute solution (10^–5^ M), **AzaBDOPV-2T** both in dilute solution (10^–5^ M) and in thin film; (b) DFT-optimized geometries of **AzaBDOPV-2T** and **BDOPV-2T** fragments. Alkyl chains were replaced by methyl groups for simplicity (geometries were optimized at the b3lyp/6-311g(d,p) level).

Cyclic voltammetry (CV) was carried out to investigate the energy levels of the monomer **AzaBDOPV** and the polymer **AzaBDOPV-2T** (Fig. S4† and 3a). After the introduction of nitrogen atoms, the LUMO level of the monomer **AzaBDOPV** reaches –4.35 eV, which is 0.11 eV lower than that of **BDOPV**, suggesting that **AzaBDOPV** might be a more efficient electron-deficient building block for n-type conjugated polymers. The HOMO/LUMO levels of **AzaBDOPV-2T** measured from CV are –5.80/–4.37 eV, which are consistent with the HOMO/LUMO levels (–5.77/–4.45 eV) estimated from photoelectron spectroscopy (PES) (Fig. S5†) and the optical bandgap. Note that the LUMO level of **AzaBDOPV-2T** is also significantly lower than that of **BDOPV-2T** (–4.15 eV), indicating that **AzaBDOPV-2T** might exhibit more efficient and stable electron transporting properties than **BDOPV-2T**.9*c* To our knowledge, polymer **AzaBDOPV-2T** is among the most electron-deficient conjugated polymers reported to date.^15^ The HOMO level of **AzaBDOPV-2T** is nearly the same as that of **BDOPV-2T** (HOMO level of –5.72 eV), indicating that the introduction of nitrogen at the position near the amide group has larger effect on the LUMO than the HOMO. This result was in accordance with the calculated molecular orbital distribution, where the HOMO of **AzaBDOPV-2T** is delocalized and the LUMO is localized on the **AzaBDOPV** cores (Fig. 3b).

**Fig. 3:**
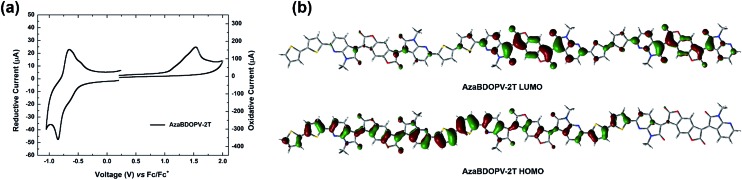
(a) Cyclic voltammogram of the polymer **AzaBDOPV-2T** in thin film prepared by drop-casting the polymer solution (1 mg mL^–1^ in CHCl_3_) on a glassy carbon electrode; (b) calculated molecular orbitals of the **AzaBDOPV-2T** trimer at the b3lyp/6-31g(d) level.

Field-effect transistors with a top-gate/bottom-contact (TG/BC) configuration were then fabricated to characterize the charge transport properties of **AzaBDOPV-2T**. The semiconducting layer was deposited by spin-coating the polymer solution (3 mg mL^–1^ in ODCB) on an Au (source–drain)/SiO_2_ substrate. *n*-Octane was then spin-coated onto the as-spun **AzaBDOPV-2T** film before thermal annealing for 5 min. Several annealing temperatures were tried, and annealing at 200 °C gave the best device performance (Fig. S6†). After thermal annealing, a CYTOP solution was spin-coated on top of the film as the dielectric layer, and an aluminum layer was thermally evaporated as the gate electrode. All devices were fabricated in a glovebox and tested in air (*R*_H_ = 50–60%). **AzaBDOPV-2T** displayed typical n-type transport characteristics (Fig. 4), with the highest electron mobility up to 3.22 cm^2^ V^–1^ s^–1^ (an average mobility of 1.63 cm^2^ V^–1^ s^–1^ from seventeen devices and the standard deviation was 0.54 cm^2^ V^–1^ s^–1^), whereas the reference polymer **BDOPV-2T** showed highest electron mobilities of up to 1.74 cm^2^ V^–1^ s^–1^.9*c* This result is attributed to the fact that **AzaBDOPV-2T** has a lower LUMO energy level and less conformational disorder than **BDOPV-2T**.

**Fig. 4:**
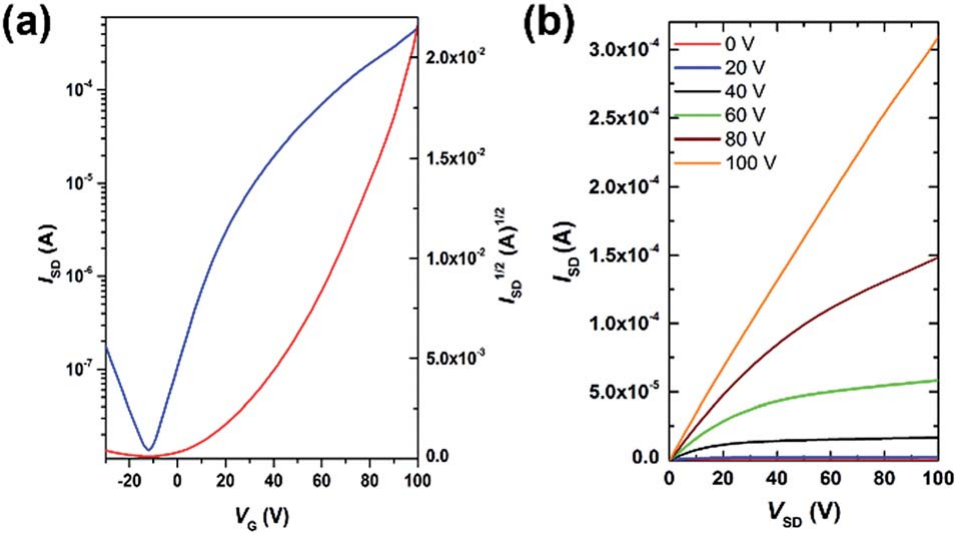
(a) Transfer and (b) output characteristics of an **AzaBDOPV-2T** device tested under ambient conditions (*L* = 5 μm, *W* = 100 μm, *C*_i_ = 3.5 nF cm^–2^), measured *V*_T_ = 40 V.

The molecular packing and surface morphology of the **AzaBDOPV-2T** film were investigated by grazing incident X-ray diffraction (GIXD) and tapping-mode atomic force microscopy (AFM) as shown in Fig. 5 and S7.† The film of **AzaBDOPV-2T** showed a strong out-of-plane diffraction peak (100) at a 2*θ* value of 2.50°, corresponding to a *d*-spacing of 28.4 Å (*λ* = 1.24 Å). Another four orders of diffraction peaks attributed to (*h*00) diffractions and an in-plane diffraction peak of (010) was also observed, indicating a distinct edge-on lamellar packing formed in the film. After the introduction of nitrogen atoms, the π–π stacking distance of **AzaBDOPV-2T** was measured to be 3.44 Å (Fig. S8†), which was shorter than that of **BDOPV-2T** (3.55 Å), suggesting stronger inter-chain interactions of **AzaBDOPV-2T** in the solid state compared to **BDOPV-2T**. The strong crystallinity of **AzaBDOPV-2T** is also supported by the AFM image, which displays a fiber-like intercalating network with obvious crystallized zones. Both the close π–π stacking distance and strong crystallinity are beneficial to the high mobilities of **AzaBDOPV-2T**.

**Fig. 5:**
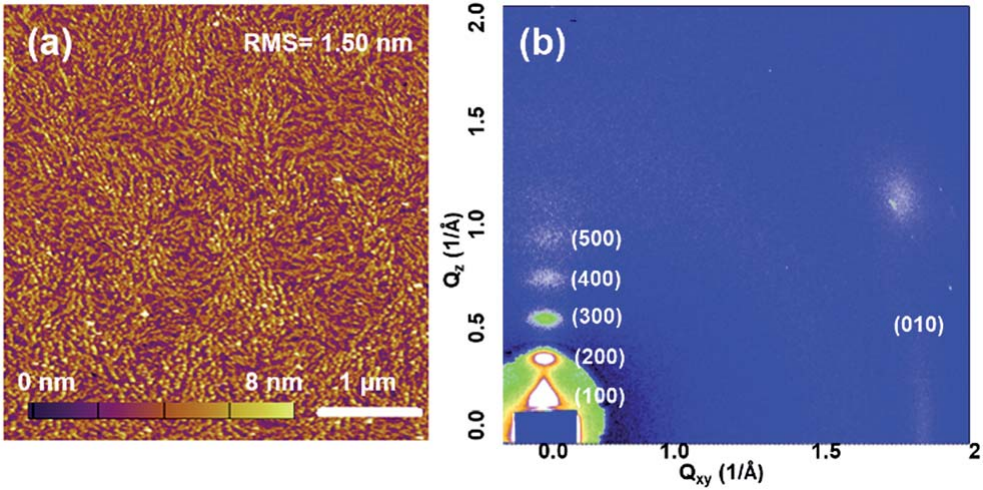
(a) AFM height image and (b) 2D-GIXD pattern of **AzaBDOPV-2T** film spin-coated on a SiO_2_ substrate after thermal annealing at 200 °C for 5 min.

## Conclusions

In conclusion, we have developed a novel electron-deficient building block, **AzaBDOPV**, which shows a much lower LUMO level compared with **BDOPV**. Based on the **AzaBDOPV** unit, the D–A conjugated polymer, **AzaBDOPV-2T**, displays a low LUMO level down to –4.37 eV and therefore typical n-type transport characteristics, with electron mobilities up to 3.22 cm^2^ V^–1^ s^–1^ in air. These investigations demonstrate that the incorporation of electron-withdrawing sp^2^-nitrogen atoms into a **BDOPV**-based polymer not only lowers the energy levels of the conjugated polymer, but also optimizes its backbone conformation, leading to improved inter-chain interactions and film microstructures, which are critical to achieving high device performance.

## Supplementary Material

Supplementary information
