# Supplementary material for: Embedding electron-deficient nitrogen atoms in polymer backbone towards high performance n-type polymer field-effect transistors
Source: Chem Sci. 2016 Jun 13;7(9):5753–7. doi: 10.1039/c6sc01380e (PMC6022156; doi:10.1039/c6sc01380e)
Supplement: Supplementary file 1 [file SC-007-C6SC01380E-s001.pdf]

Electronic Supplementary Material (ESI) for Chemical Science.
This journal is © The Royal Society of Chemistry 2016

Supporting Information

For

Embedding Electron-Deficient Nitrogen Atoms in Polymer Backbone towards High Performance *n*-Type Polymer Field-Effect Transistors

Ya-Zhong Dai, Na Ai, Yang Lu, Yu-Qing Zheng, Jin-Hu Dou, Ke Shi, Ting Lei, Jie-Yu Wang, and Jian Pei*

Beijing National Laboratory for Molecular Sciences, Key Laboratory of Bioorganic Chemistry and Molecular Engineering of Ministry of Education, Key Laboratory of Polymer Chemistry and Physics of Ministry of Education, Center for Soft Matter Science and Engineering, College of Chemistry and Molecular Engineering, Peking University, Beijing 100871, China

jianpei@pku.edu.cn (J. P.)

Table of Contents

1. General procedures and experimental details S2

2. Figures S1-S8 .... S3

3. Synthetic procedures and characterization S5

4. ^{1}H and ^{13}C NMR spectra S8

5. Reference of Computational Studies S13

1. General procedures and experimental details

Chemical reagents and CYTOP were purchased and used as received. All air and water sensitive reactions were performed under nitrogen atmosphere. Toluene and tetrahydrofuran (THF) were distilled from sodium prior to use. ^{1}H and ^{13}C NMR spectra were recorded on Bruker ARX-400 (400 MHz). All chemical shifts were reported in parts per million (ppm). ^{1}H NMR chemical shifts were referenced to TMS (0 ppm), and ^{13}C NMR chemical shifts were referenced to $CDCl_3$ (77.00 ppm). Mass spectra were recorded on a Bruker BIFLEX III mass spectrometer. Elemental analyses were performed using a German Vario EL III elemental analyzer. Thermal gravity analyses (TGA) were carried out on a TA Instrument Q600 analyzer, and differential scanning calorimetry (DSC) analyses were performed on a METTLER TOLEDO Instrument DSC822 calorimeter. Gel permeation chromatography (GPC) was performed on Polymer Laboratories PL-GPC220 at 150 ^{o}C using 1,2,4-tricholorobenzene (TCB) as eluent. Absorption spectra were recorded on PerkinElmer Lambda 750 UV-*vis* spectrometer. Cyclic voltammetry (CV) was performed on BASI Epsilon workstation. Thin film measurements were carried out in acetonitrile containing 0.1 M *n*-Bu_4NPF_6 as a supporting electrolyte. Glassy carbon electrode was used as a working electrode and a platinum wire as a counter electrode, and all potentials were recorded versus Ag/AgCl (saturated) as a reference electrode (scan rate: 50 mV s^{-1}). Photo-electron spectra (PES) were performed on AC-2 photoelectron spectrometer (Riken-Keiki Co.). The X-ray diffraction data were obtained at beamline BL14B1 of the Shanghai Synchrotron Radiation Facility (SSRF) at a wavelength of 1.2398 Å. BL14B1 is a beamline based on bending magnet and a Si (111) double crystal monochromator was employed to monochromatize the beam. The size of the focus spot is about 0.5 mm and the end station is equipped with a Huber 5021 diffractometer. NaI scintillation detector was used for data collection. Atomic force microscopy studies were performed with a Nanoscope IIIa microscope (Extended Multimode). All experiments were carried out in tapping mode under ambient conditions. A silicon nitride cantilever (Budget Sensors Tap300Al) was used with a resonant frequency around 300 kHz.

Device fabrication and characterization. Glass slides with patented gold electrodes (source-drain) were used as substrates, after being sequentially cleaned in an ultrasonic bath with acetone, detergent, deionized water, and isopropanol for 10 min each. The polymer **AzaBDOPV-2T** was dissolved in dichlorobenzene (Aldrich), 3 mg mL^{-1} and filtered using a 0.45 μm polytetrafluoroethylene filter. FETs with top-gate/bottom-contact (TG/BC) configuration were then fabricated by spin-coating the polymer solution onto the substrate (1500 rpm, 90 s) in a N_2 glove box. A variety of solvents, including hexane, *n*-octane and *n*-dodecane were spin-coated onto the as-spun **AzaBDOPV-2T** films before the films were thermal annealed at optimized temperature (200 ^{o}C) for 5 min and *n*-octane was finally chosen to give the best device performance. A fluoropolymer (CYTOP) solution was spin-coated on top of the film as the dielectric layer, and the transistors were completed by thermal evaporating an aluminum layer as the gate electrode. The evaluations of the FETs were carried out in atmosphere (humidity 50-60%) on a probe stage using a Keithley 4200 SCS as parameter analyzer. The carrier mobility, μ, was calculated from the data in the saturated regime according to the equation $I_{SD} = (W/2L)C_i\mu(V_G - V_T)^2$, where I_{SD} is the

drain current in the saturated regime. *W* and *L* are the semiconductor channel width and length, respectively. C_i (C_i = 3.7 nF) is the capacitance per unit area of the gate dielectric layer. V_G and V_T are the gate voltage and threshold voltage, respectively. $V_G - V_T$ of the device was determined from the relationship between the square root of I_{SD} and V_G at the saturated regime.

2. Figures S1-S8

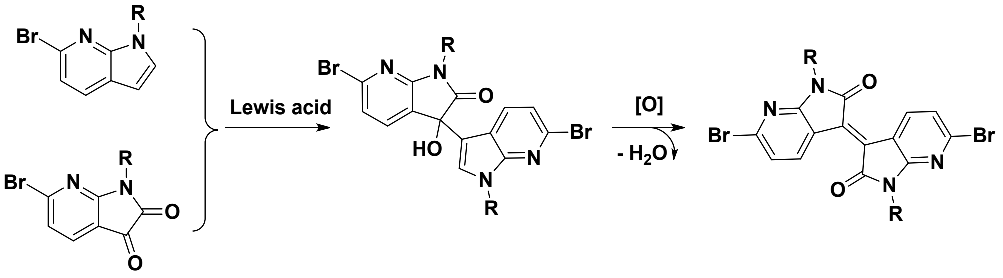

Figure S1. Proposed pathway for in situ generation of 7,7'-azaindigo.

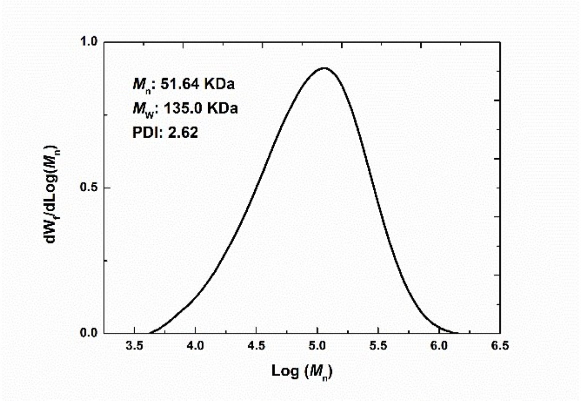

Figure S2. Gel permeation chromatography (GPC) trace of **AzaBDOPV-2T**. Molecular weight was evaluated with 1,2,4-tricholorobenzene as eluent at 150 °C.

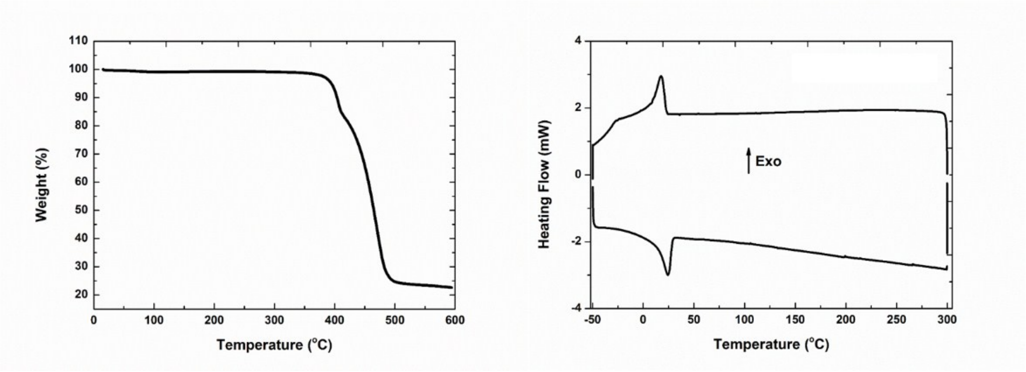

Figure S3. (a) Thermal gravity analysis (TGA) of **AzaBDOPV-2T** (5% loss, 390 °C); (b) Differential scanning calorimetry (DSC) trace of **AzaBDOPV-2T** (*T* = 25 °C). *T* is the phase transition temperature caused by the melting of the long alkyl chains.

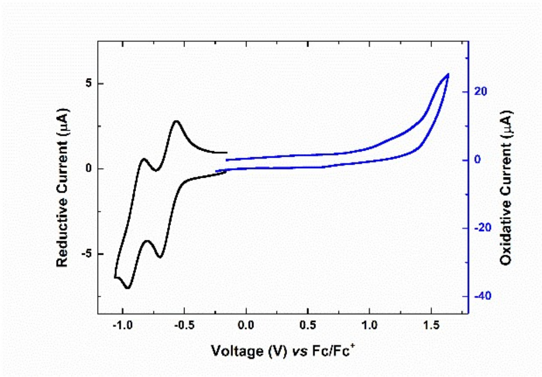

Figure S4. Cyclic voltammogram of the monomer **AzaBDOPV** in solution (1 mg/mL in DCB).

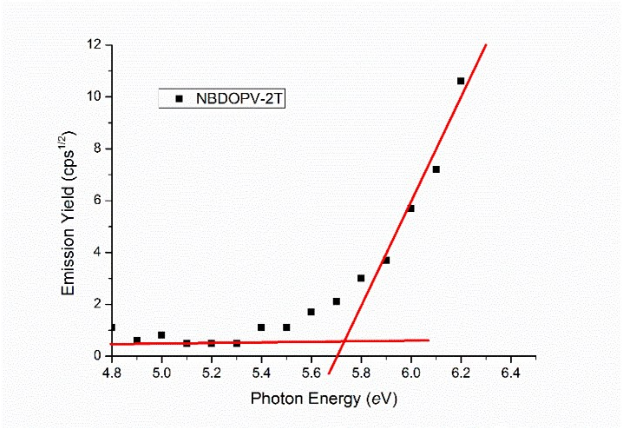

Figure S5. Photo-electron spectrum (PES) of **AzaBDOPV-2T** in thin film (Work function: 5.77 eV).

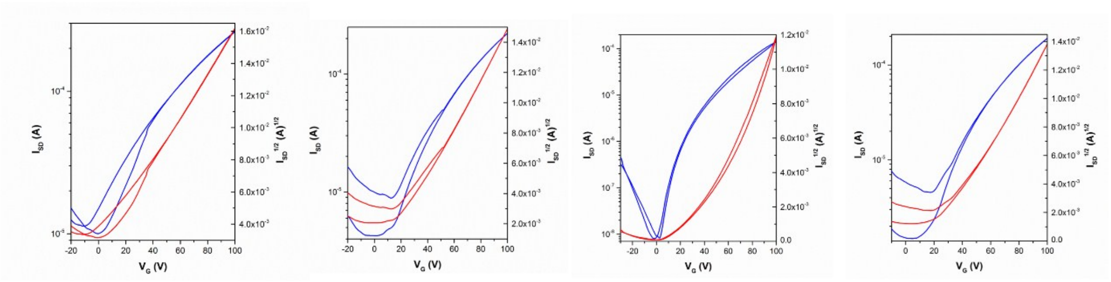

Not annealed **180 °C** **200 °C** **220 °C**

Figure S6. The transfer curves of AzaBDOPV-2T annealed different temperatures.

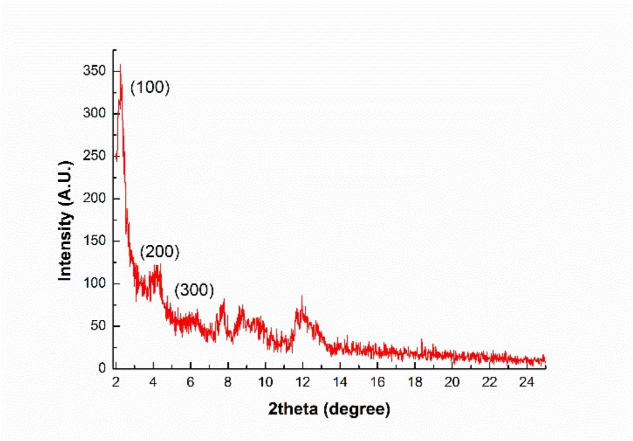

Figure S7. Out-of-plane GIXD plot of **AzaBDOPV-2T** film obtained from a point detector in beamline BL14B1 (SSRF).

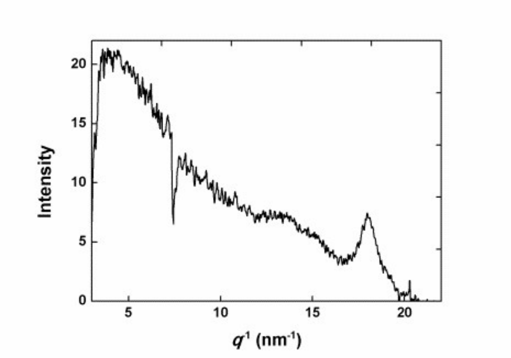

Figure S8. In-plane GIXD plot of AzaBDOPV-2T film.

3. Synthetic procedures and characterization

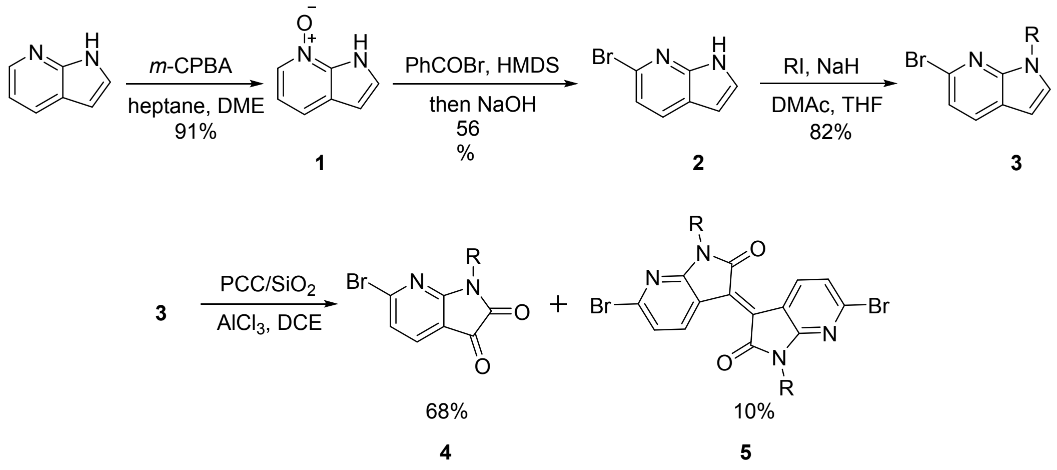

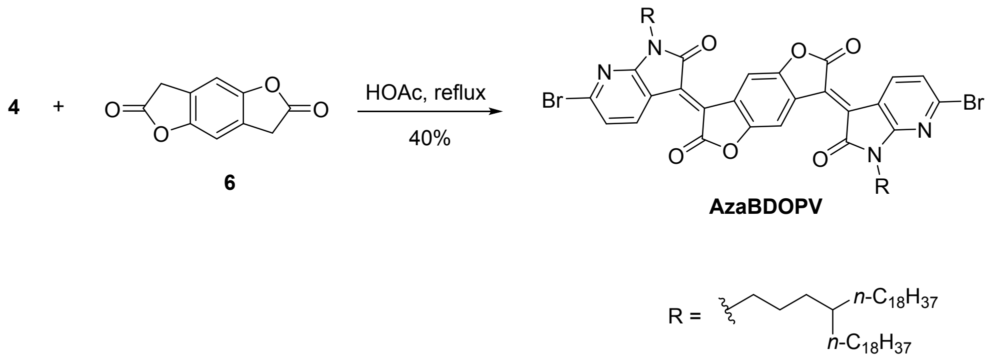

Compound 1 and **6** was synthesized according to the literature.[1,2] RI is commercially available from Lyn (Beijing) Science & Technology Co., Ltd. (http://www.lyntech.cn/).

Compound 2: To a solution of **1** (420 mg, 3.1 mmol) in a mixure of THF (30 mL) and hexamethyldisilazane (501 mg, 3.1 mmol), benzoyl bromide (1.15 g, 6.2 mmol) was slowly added. The mixture was then stirred at room temperature for 3 h and then filtered through a celite pad. The solvents were removed under reduced pressure and the residue was directly dissolved in MeOH (50 mL) with NaOH (124 mg ,3.1mmol). After stirring at room temperature for 12 h, MeOH was removed and the residue was extracted with EtOAc and concentrated under reduced pressure to give **2** as a white solid (342 mg, 56 %). ^{1}H NMR (400 MHz, $CDCl_3$, ppm): *δ* 10.97 (s, 1H), 7.83 (d, *J* = 8.1 Hz, 1H), 7.41 (dd, *J* = 3.3, 2.6 Hz, 1H), 7.25 (d, *J* = 8.1 Hz, 1H), 6.51 (dd, *J* = 3.3, 2.0 Hz, 1H). ^{13}C NMR (126 MHz, DMSO-d_6, ppm): *δ* 148.17, 133.87, 131.61, 127.17, 119.18, 119.08, 100.79. ESI-HRMS calcd. for $[M + H]^+$: 196.9714; Found: 196.9711.

Compound 3: To a solution of **2** (1.031 g, 5.23 mmol) in a mixture of THF (30 mL) and *N*,*N*'-dimethylformamide (DMAc) (30 mL), RI (3.583 g, 5.20 mmol) and NaH (0.610 g, 25.4 mmol) was added. The mixture was stirred at 25 °C for 8 h and then quenched by water. The solvents were removed under reduced pressure. The residue was dissolved in CH_2Cl_2 (100 mL) and then washed with water and brine, and dried with Na_2SO_4. After removal of the solvents under reduced pressure, the residue was purified by silica gel chromatography with eluent (PE:CH_2Cl_2 = 10:1) to give **3** as a white solid (3.25 g, 82 %). ^{1}H NMR (400 MHz, $CDCl_3$, ppm): *δ* 7.72 (d, *J* = 8.1 Hz, 1H), 7.21–7.14 (m, 2H), 6.43 (d, *J* = 3.4 Hz, 1H), 4.22 (t, *J* = 7.2 Hz, 2H), 1.79–1.86 (m, 2H), 1.18–1.30 (m, 71H), 0.90–0.86 (t, J = 6.8 Hz, 6H). ^{13}C NMR (126 MHz, $CDCl_3$, ppm): *δ* 147.07, 134.55, 130.67, 127.91, 119.17, 119.12, 99.68, 45.0, 33.5, 31.9, 30.0, 29.7, 29.7, 29.4, 27.4, 26.6, 22.7, 14.1. Elemental Anal. Calcd: for $C_{47}H_{85}BrN_2$: C, 74.46; H, 11.30; N, 3.70; Found: C, 74.47; H, 11.42; N, 3.68. ESI-HRMS calcd. for $[M + H]^+$: 757.5974; Found: 757.5969.

Compound **4** and **5:** To a round-bottom-flask containing PCC (10.8 g, 0.05 mol) in dry dichloroethane (DCE) (250 mL) was added compound **3** (15.16 g, 0.02 mol) while stirring at room temperature. Then $AlCl_3$ (267 mg, 0.002 mol) was added to the resulting orange suspension solution, and the reaction mixture was stirred at 90 °C for 8 h. After filtration to remove insoluble substance and removal of the solvent, the residue was purified by

chromatography on silica gel with eluent (PE:CH_2Cl_2 = 4:1) to give the respective product. Compound **4**: ^{1}H NMR (400 MHz, $CDCl_3$, ppm): δ 7.63 (d, J = 7.7 Hz, 1H), 7.27 (d, J = 7.7 Hz, 1H), 3.80 (t, J = 7.4 Hz, 2H), 1.73 (m, 2H), 1.22–1.30 (m, 71H), 0.86–0.89 (t, J = 6.8 Hz, 6H). ^{13}C NMR (126 MHz, $CDCl_3$, ppm): δ 180.91, 164.23, 158.12, 150.18, 133.94, 123.26, 110.32, 39.7, 37.4, 33.6, 33.3, 32.0, 30.2, 29.7, 29.4, 27.9, 26.7, 24.1, 22.7, 14.1. Elemental Anal. Calcd: for $C_{47}H_{83}BrN_2O_2$: C, 71.63; H, 10.62; N, 3.55; Found: C, 71.67; H, 10.76; N, 3.45. ESI-HRMS calcd. for $[M + H]^+$: 787.5716; Found: 787.5711.
Compound **5**: ^{1}H NMR (400 MHz, $CDCl_3$, ppm): δ 9.31 (d, J = 8.3 Hz, 2H), 7.19 (d, J = 8.3 Hz, 2H), 3.85 (t, J = 7.3 Hz, 4H), 1.70–1.72 (m, 4H), 1.18–1.30 (m, 142H), 0.86–0.89 (t, J = 6.8 Hz, 12H). ^{13}C NMR (101 MHz, $CDCl_3$, ppm): δ 167.48, 157.85, 143.86, 139.10, 131.43, 121.91, 114.48, 40.3, 37.3, 33.8, 32.2, 30.9, 30.4, 30.0, 30.0, 29.7, 29.6, 27.5, 26.9, 24.9, 23.0, 14.4. MALDI-HRMS calcd. for $[M + H]^+$: 1545.1469; Found: 1545.1468.

AzaBDOPV: To a solution of **4** (1.025g,1.3 mmol) in acetic acid (10 mL), **6** (101 mg, 0.53 mmol) and toluenesulfonic acid monohydrate (28 mg, 0.15 mmol) was added. After reflux under argon atmosphere for 12 h, the mixture was cooled to room temperature and filtered. After washed with acetic acid and methanol, the filtration residue was dissolved in $CHCl_3$ (100 mL) and then washed with water and brine, and dried with Na_2SO_4. After removal of the solvents under reduced pressure, the residue was purified by silica gel chromatography with eluent (PE:$CHCl_3$ = 1:1) to give **AzaBDOPV** as a dark blue solid (367 mg, 40%). ^{1}H NMR (400 MHz, $CDCl_3$, ppm): δ 9.19 (s, 2H), 9.11 (d, J = 8.4 Hz, 2H), 7.22 (d, J = 8.4 Hz, 2H), 3.91–3.85 (t, 4H), 1.75 (m, 4H), 1.24–1.31 (m, 142H), 0.86–0.89 (t, J = 6.8 Hz, 12H).. ^{13}C NMR (126 MHz, $CDCl_3$, ppm): δ 166.98, 166.67, 158.52, 152.33, 145.39, 138.99, 133.92, 127.69, 126.97, 122.28, 114.10, 111.45, 39.8, 37.4, 33.7, 33.3, 32.0, 30.2, 29.7, 29.7, 29.4, 28.0, 26.7, 24.1, 22.7, 14.1. Elemental Anal. Calcd: for $C_{104}H_{168}N_4O_6$: C, 72.19; H, 9.79; N, 3.24; Found: C, 72.21; H, 9.52; N, 3.16. MALDI-TOF MS: 1730.5 $[M]^+$

AzaBDOPV-2T: **AzaBDOPV** (50 mg, 0.0289 mmol), 5,5'-bis(trimethylstannyl)-2,2'-bithiophene (14.2 mg, 0.0289 mmol), $Pd_2(dba)_3$ (0.5 mg, 2 mol%.), P(o-tol)$_3$ (0.7 mg, 8 mol%), and 10 mL of toluene were added to a Schlenk tube. The tube was charged with nitrogen through a freeze-pump-thawcycle for three times. The mixture was stirred for 12 h at 120 °C. *N*,*N*'-Diethylphenylazothioformamide (10 mg) was then added and then the mixture was stirred for 0.5 h to remove any residual catalyst before being precipitated into methanol (200 mL). The precipitate was filtered through a nylon filter and purified via Soxhlet extraction for 6 h with acetone, 6 h wi th hexane, and finally collected with chloroform. The chloroform solution was then concentrated by evaporation and precipitated into methanol (250 mL) and filtered off to afford a dark solid (47 mg, yield: 94%). Elemental Anal. calcd: for $(C_{112}H_{172}N_4O_6S_2)_n$: C, 77.55; H, 9.99; N, 3.23; found: C, 75.87; H, 10.02; N, 3.13.

References：

(1) Schneller, S. W.; Luo, J.-K. *J. Org. Chem.* **1980**, *45*, 4045;
(2) Lei, T.; Dou, J.-H.; Cao, X.-Y.; Wang, J.-Y.; Pei, J. *J. Am. Chem. Soc.* **2013**, *135*, 12168.

4. ^{1}H and ^{13}C NMR spectra

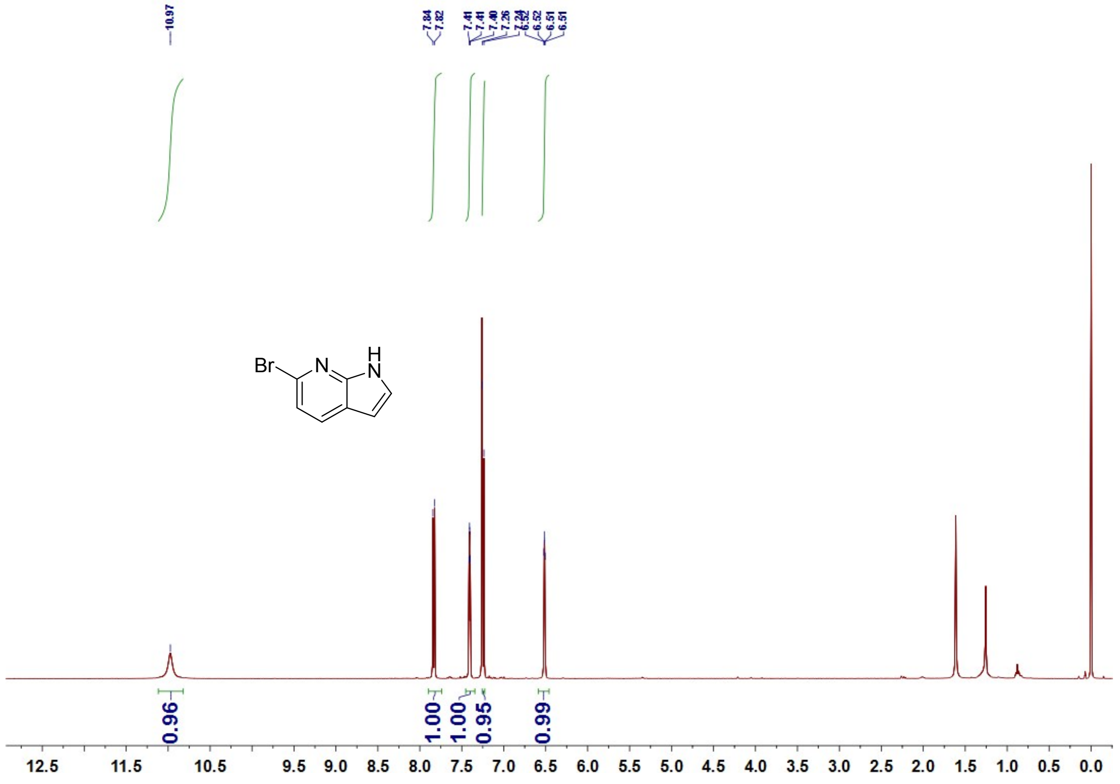

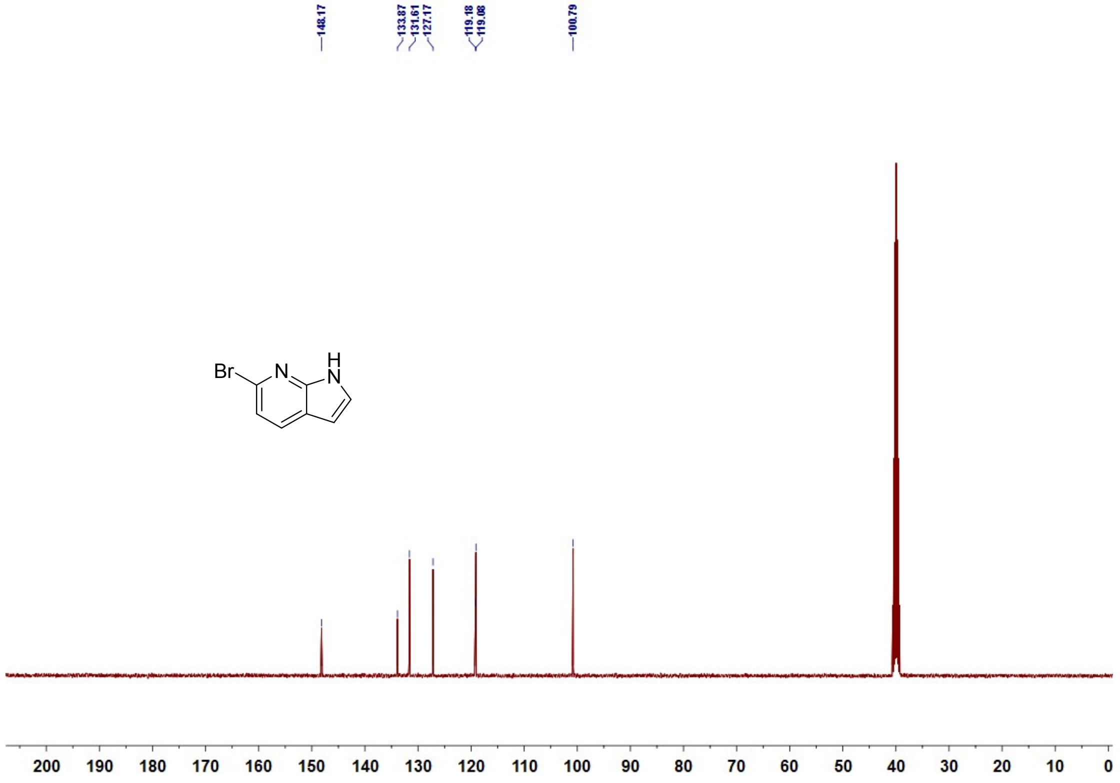

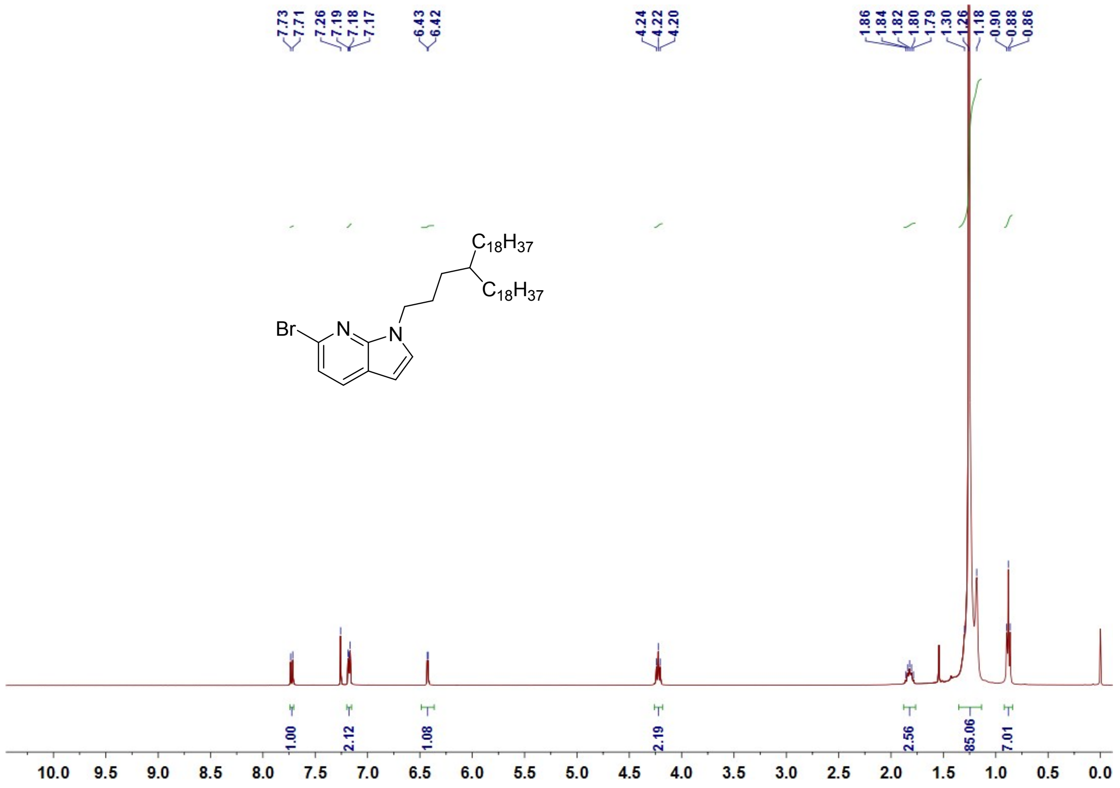
7.73
7.71
7.26
7.19
7.18
7.17
6.43
6.42
4.24
4.22
4.20
1.86
1.84
1.82
1.80
1.79
1.30
1.26
1.18
0.90
0.88
0.86
C18H37
C18H37
Br
N
N
1.00
2.12
1.08
2.19
2.56
85.06
7.01
10.0 9.5 9.0 8.5 8.0 7.5 7.0 6.5 6.0 5.5 5.0 4.5 4.0 3.5 3.0 2.5 2.0 1.5 1.0 0.5 0.0

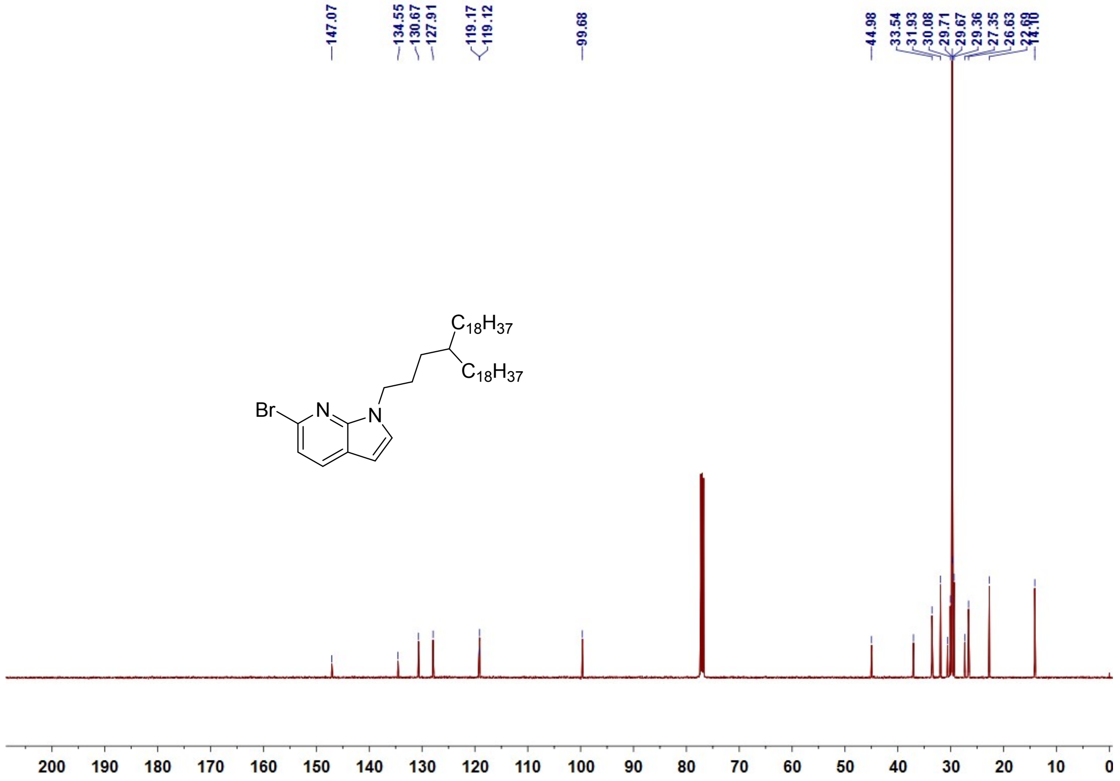
147.07
134.55
130.67
127.91
119.17
119.12
99.68
44.98
33.54
31.93
30.08
29.71
29.67
29.36
27.35
26.63
C18H37
C18H37
Br
N
N
200 190 180 170 160 150 140 130 120 110 100 90 80 70 60 50 40 30 20 10 0

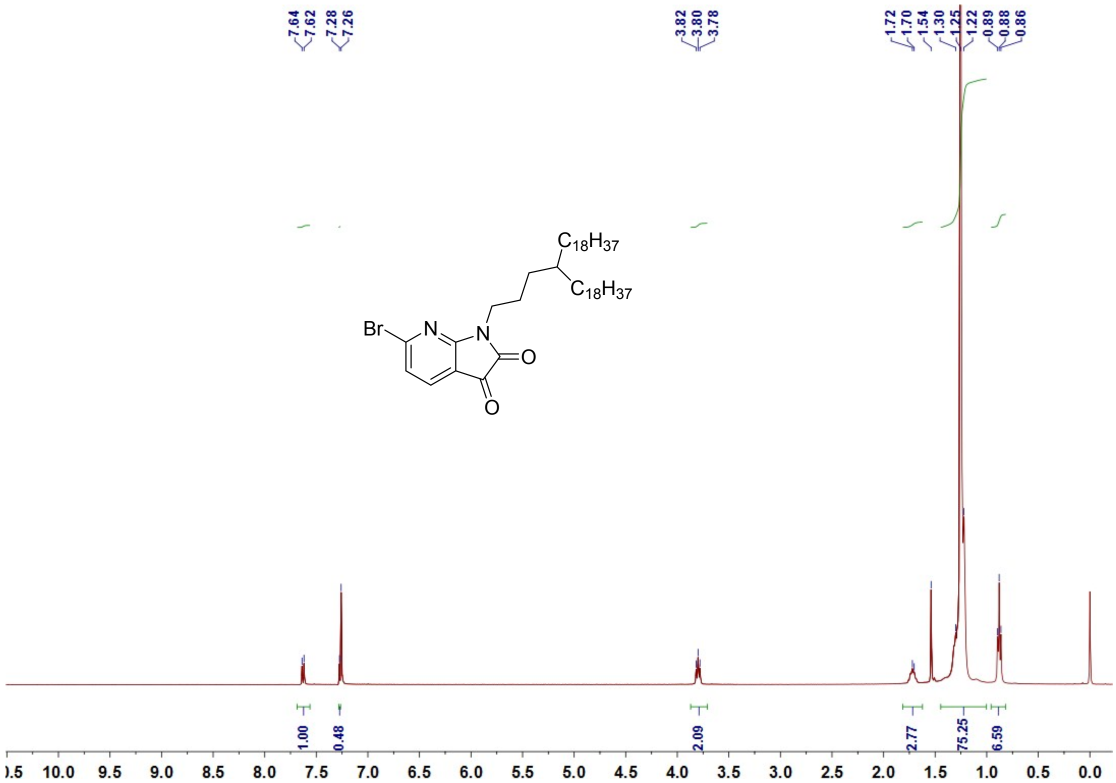
7.64
7.62
7.28
7.26
3.82
3.80
3.78
1.72
1.70
1.54
1.30
1.25
1.22
0.89
0.88
0.86
C18H37
C18H37
Br
N
N
O
O
1.00
0.48
2.09
2.77
75.25
6.59
0.5 10.0 9.5 9.0 8.5 8.0 7.5 7.0 6.5 6.0 5.5 5.0 4.5 4.0 3.5 3.0 2.5 2.0 1.5 1.0 0.5 0.0

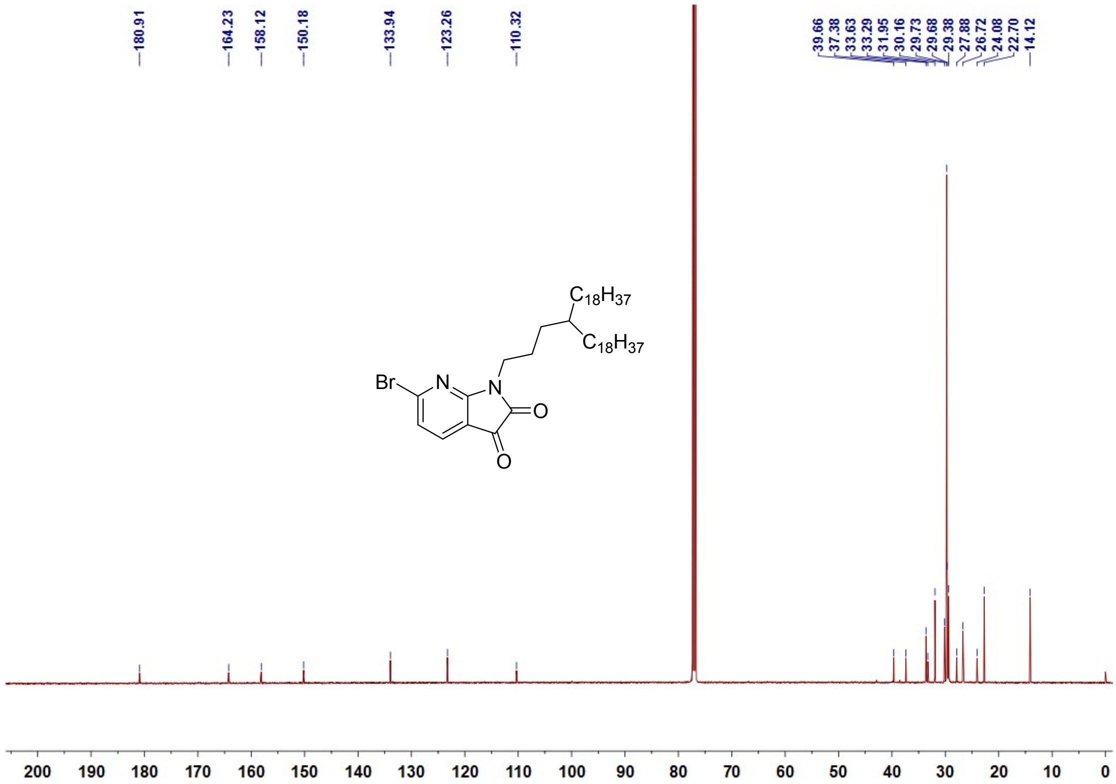
180.91
164.23
158.12
150.18
133.94
123.26
110.32
39.66
37.38
33.63
33.29
31.95
30.16
29.73
29.68
29.38
27.88
26.72
24.08
22.70
14.12
C18H37
C18H37
Br
N
N
O
O
200 190 180 170 160 150 140 130 120 110 100 90 80 70 60 50 40 30 20 10 0

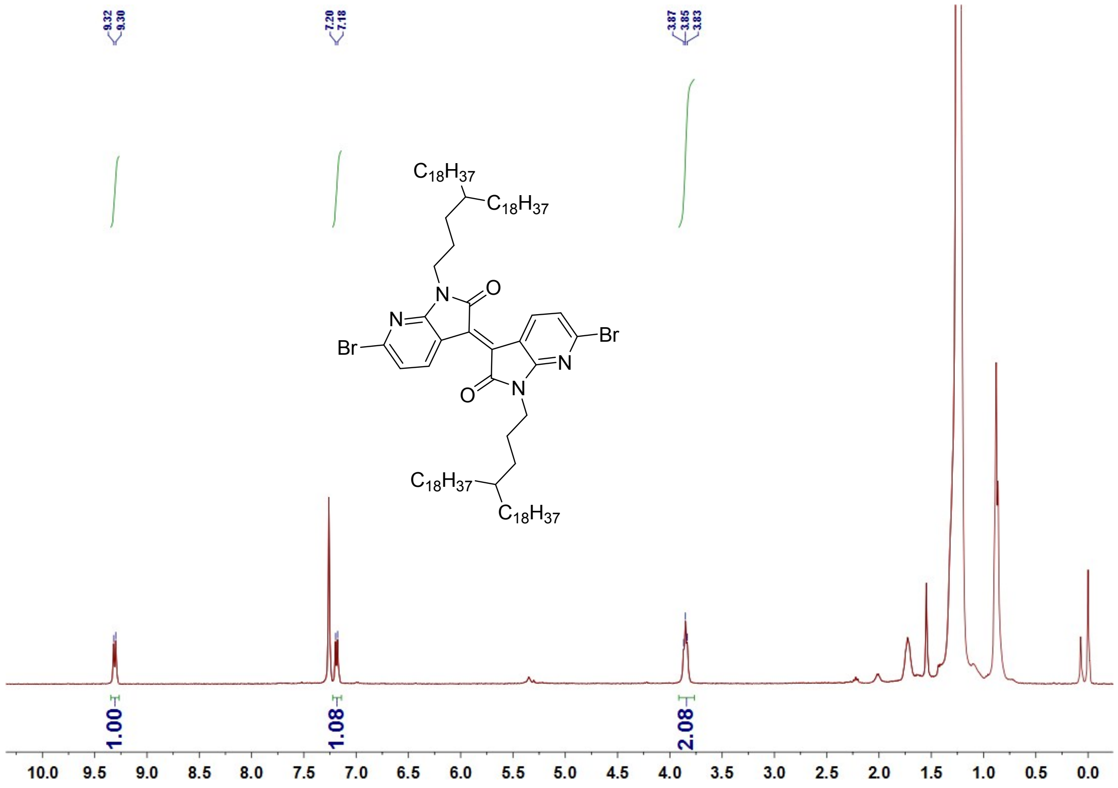

9.32
9.30
7.20
7.18
3.87
3.85
3.83
C18H37
C18H37
N
O
N
Br
Br
N
O
N
C18H37
C18H37
1.00
1.08
2.08
10.0 9.5 9.0 8.5 8.0 7.5 7.0 6.5 6.0 5.5 5.0 4.5 4.0 3.5 3.0 2.5 2.0 1.5 1.0 0.5 0.0

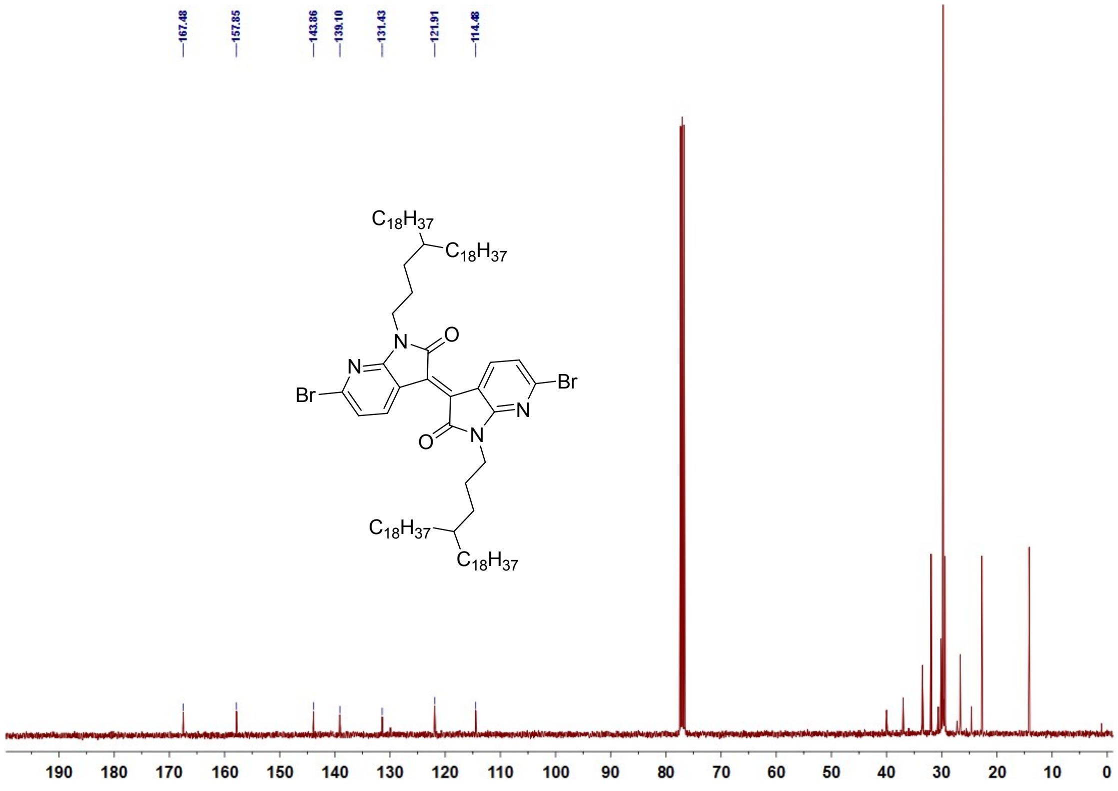

167.48
157.85
143.86
139.10
131.43
121.91
114.48
C18H37
C18H37
N
O
N
Br
Br
N
O
N
C18H37
C18H37
190 180 170 160 150 140 130 120 110 100 90 80 70 60 50 40 30 20 10 0

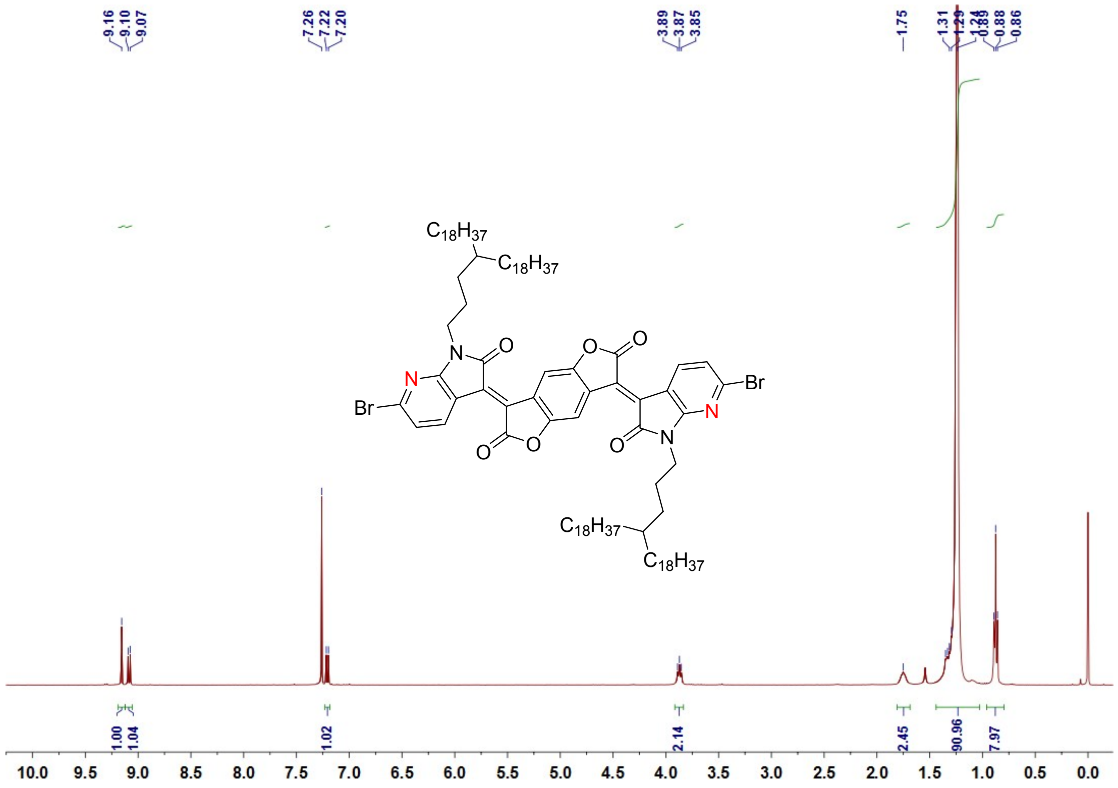

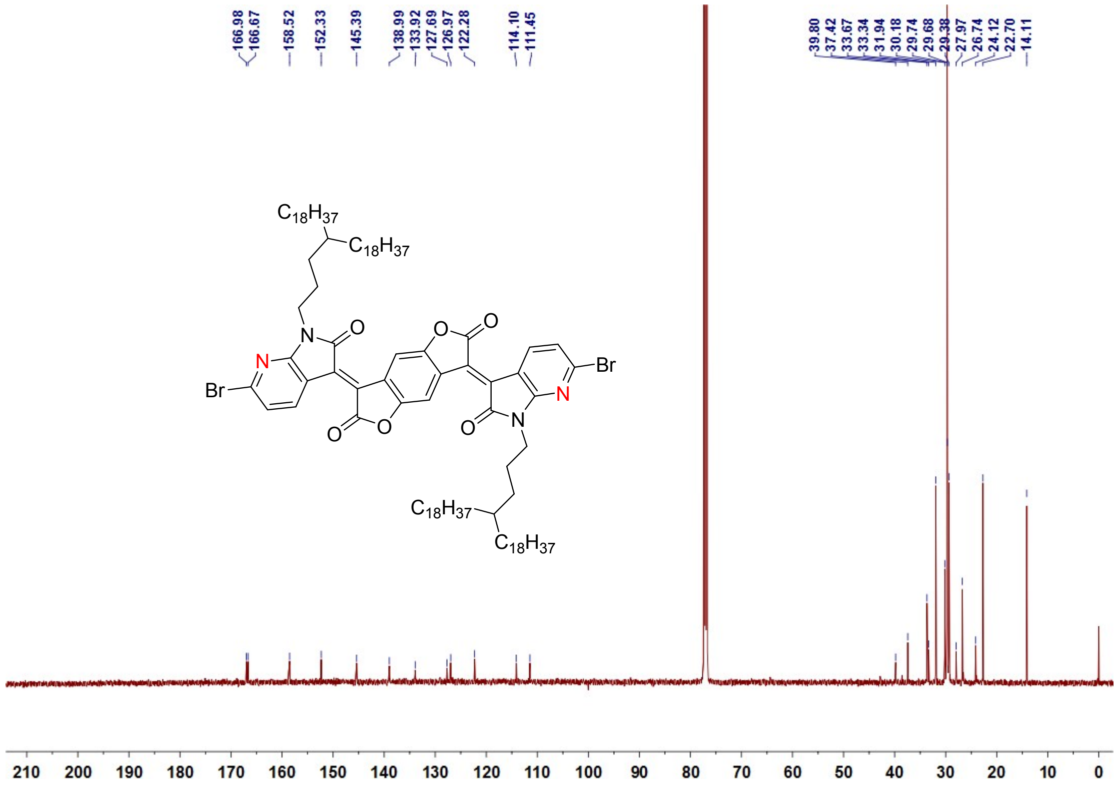

^{1}H NMR spectrum of the polymer **AzaBDOPV-2T** in 1,2-dichlorobenzene-d4:

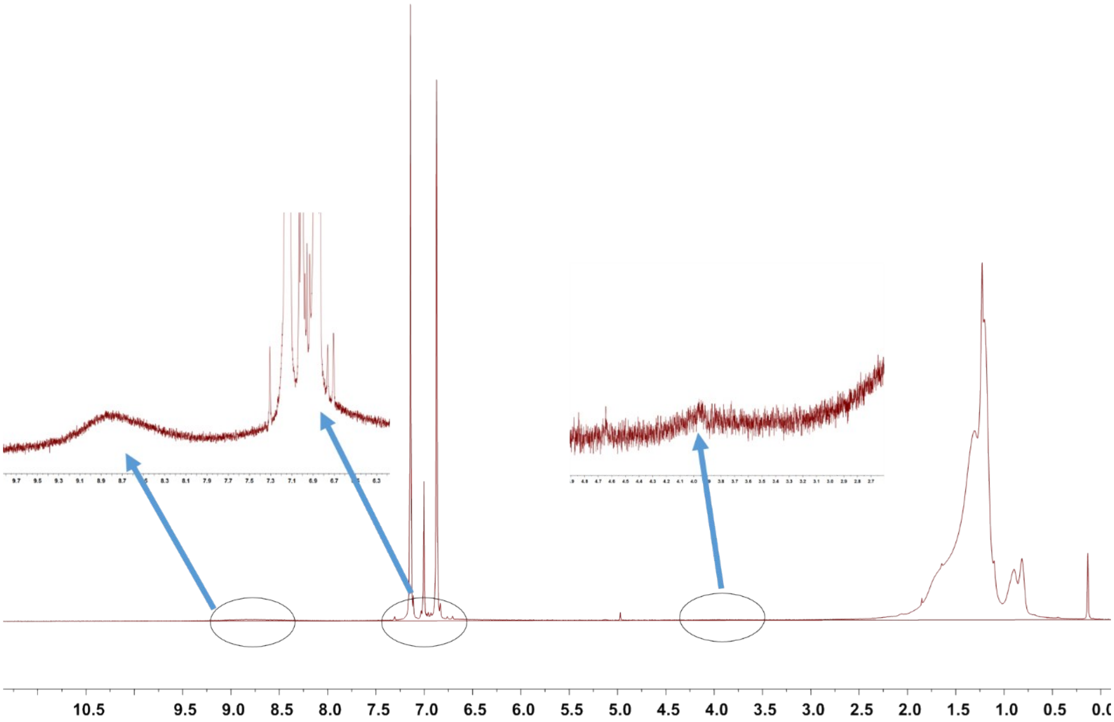

5. Reference of computational studies

(1) Gaussian 09, Revision B.01, Frisch, M. J.; Trucks, G. W.; Schlegel, H. B.; Scuseria, G. E.; Robb, M. A.; Cheeseman, J. R.; Scalmani, G.; Barone, V.; Mennucci, B.; Petersson, G. A.; Nakatsuji, H.; Caricato, M.; Li, X.; Hratchian, H. P.; Izmaylov, A. F.; Bloino, J.; Zheng, G.; Sonnenberg, J. L.; Hada, M.; Ehara, M.; Toyota, K.; Fukuda, R.; Hasegawa, J.; Ishida, M.; Nakajima, T.; Honda, Y.; Kitao, O.; Nakai, H.; Vreven, T.; Montgomery, Jr., J. A.; Peralta, J. E.; Ogliaro, F.; Bearpark, M.; Heyd, J. J.; Brothers, E.; Kudin, K. N.; Staroverov, V. N.; Kobayashi, R.; Normand, J.; Raghavachari, K.; Rendell, A.; Burant, J. C.; Iyengar, S. S.; Tomasi, J.; Cossi, M.; Rega, N.; Millam, N. J.; Klene, M.; Knox, J. E.; Cross, J. B.; Bakken, V.; Adamo, C.; Jaramillo, J.; Gomperts, R.; Stratmann, R. E.; Yazyev, O.; Austin, A. J.; Cammi, R.; Pomelli, C.; Ochterski, J. W.; Martin, R. L.; Morokuma, K.; Zakrzewski, V. G.; Voth, G. A.; Salvador, P.; Dannenberg, J. J.; Dapprich, S.; Daniels, A. D.; Farkas, Ö.; Foresman, J. B.; Ortiz, J. V.; Cioslowski, J.; Fox, D. J. Gaussian, Inc., Wallingford CT, 2010.
